# COVID-19 pneumonia and the masquerades

**DOI:** 10.1259/bjrcr.20200067

**Published:** 2020-06-24

**Authors:** Cheng Xie, Maria Tsakok, Samuel Channon-Wells, Fiona MacLeod, Heiko Peschl, Louise Wing, Rachel Benamore, Fergus Gleeson

**Affiliations:** 1 Department of Radiology, Churchill Hospital, Oxford University Hospital Trust, Old Road, Headington, Oxford, OX3 7LE, UK

## Abstract

During the COVID-19 pandemic, chest CT is frequently used to help with the diagnosis. The classic CT patterns of COVID-19 pneumonia are well-published and recognised among radiologists. However, when there are pre-existing conditions particularly in the elderly population that could mask or result in similar patterns of disease, then the diagnosis is more difficult. This imaging essay highlights the commonly encountered situations including patients with heart failure, other possible infections particularly in the immunodeficient, and when there is trauma to the thorax. We illustrate imaging clues available to the radiologist to either make the diagnosis or at least reduce the differential diagnosis

## Introduction

As the COVID-19 pandemic sweeps across the globe, early diagnosis is one of the main ways to combat the infection. The reverse-transcription polymerase chain reaction (RT-PCR) taken from nasopharyngeal swab samples has become the gold-standard in making the diagnosis. However, it is well-known that although the RT-PCR assay is highly specific, it has a low sensitivity.^1,2^ Chest CT is not a replacement for RT-PCR, but it could be used in highly suspicious cases of COVID-19 pneumonia who are severely ill or when RT-PCR is not readily available.^3^ There is also increasing evidence that chest CT is a more sensitive diagnostic tool compared to the RT-PCR assay. But, the main limitation of chest CT is its low specificity.^4^ Radiologists are often asked to provide their expertise in these situations. The imaging diagnosis is simple when the pattern of disease on the chest CT is classic of the COVID-19 pneumonia. In situations, patients with pre-existing thoracic diseases which is often the case in the at-risk elderly population, then the appearances may no longer be as classical. In this case series, we present clinical scenarios in which the diagnosis is challenging and use imaging clues available to the radiologist to either make the diagnosis or at least reduce the differential diagnoses. All the CT positive/negative cases included in this review are supported by corresponding RT-PCR test results unless specifically indicated in individual cases.

### Classic and probable COVID-19 CT patterns

**Figure 1.**
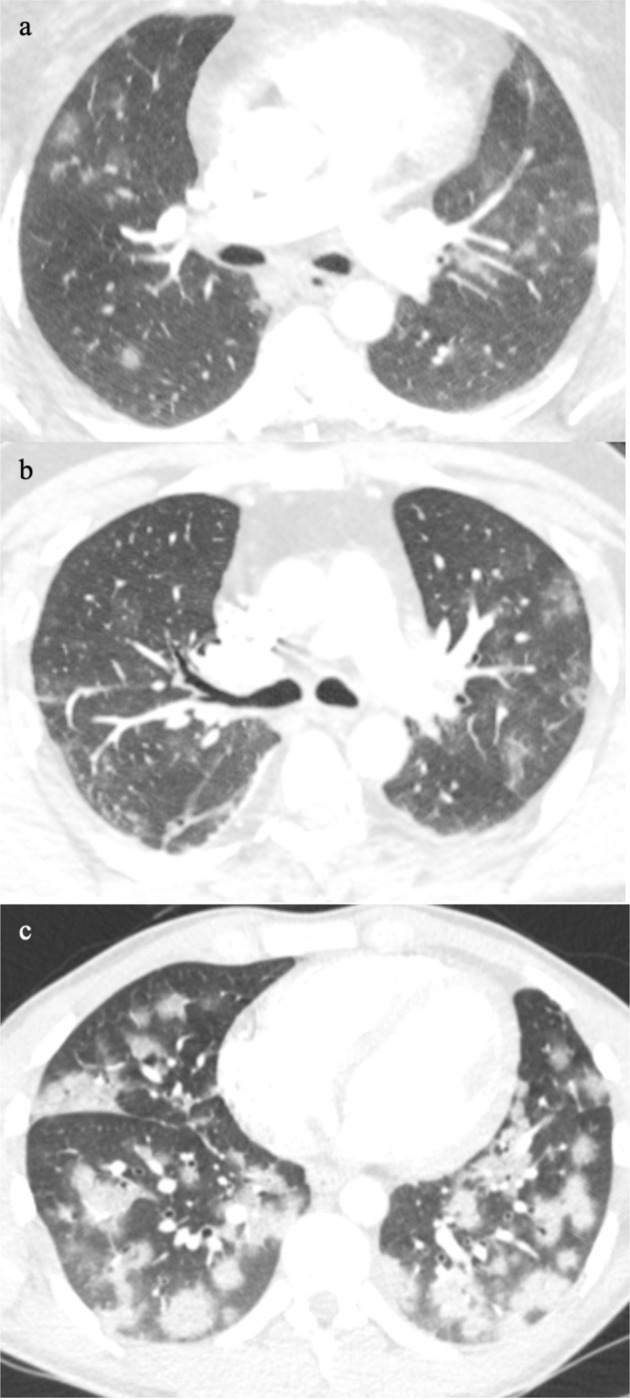
Classic multifocal pattern of COVID-19 pneumonia from minor small foci (a), ill-defined ground glass patches (b), to dense ground glass foci in a random distribution (c).

**Figure 2.**
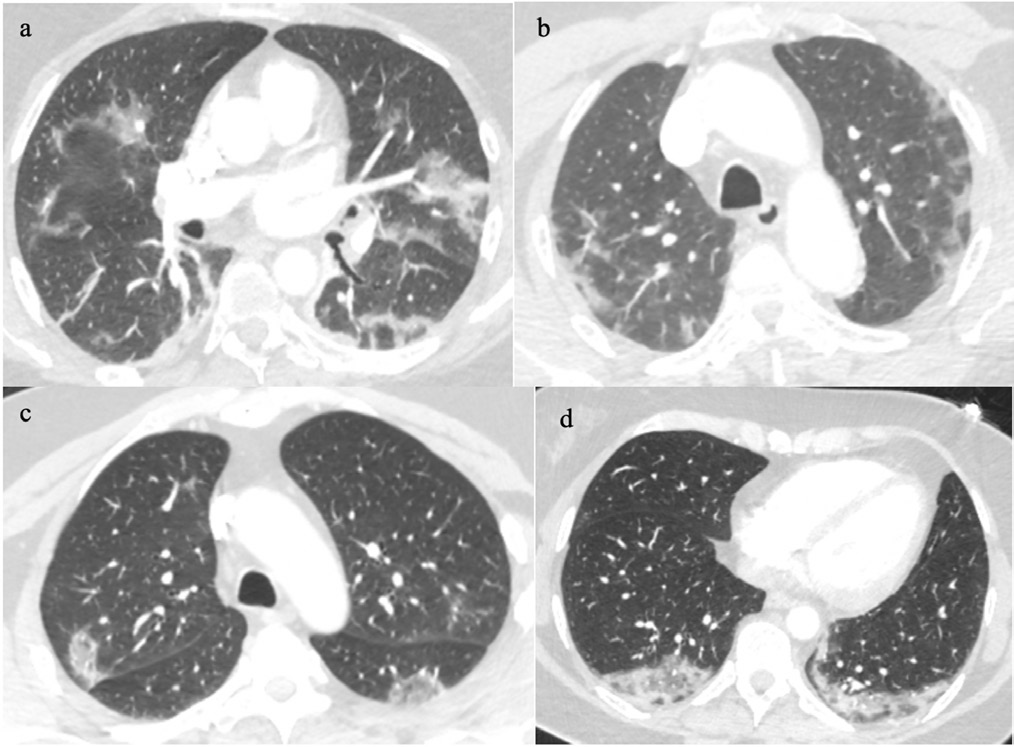
Classic COVID-19 pneumonia patterns that mimic organising pneumonia such as peripheral perilobular ground glass opacification (2a&b), and reverse halo signground glass opacification (2c&d).

Using the British Society of Thoracic Imaging (BSTI) guidelines for reporting CT scans in COVID-19 pneumonia, the classic CT manifestation of the disease is lower lobe or peripheral predominant with multiple, bilateral foci of ground glass opacification; probable pattern consists of lower lobe predominant mix of bronchocentric and peripheral consolidation with limited ground glass opacification; and indeterminate cases occurs in the context when the clinical information suggests an alternative diagnosis.^3^ From our experience, the bilateral multifocal ground glass opacification may range from minor small foci (Figure 1a), ill-defined ground glass patches (Figure 1b), to dense ground glass foci in a random distribution (Figure 1c). The diagnosis may also be made with confidence when there are patterns that mimic organising pneumonia such as peripheral perilobular ground glass opacification (Figure 2a and b), and the reverse halo sign (Figure 2c and d). These imaging patterns along with the clinical history, signs and symptoms provide the reporting radiologist with a high degree of confidence in making the diagnosis of COVID-19 pneumonia in the absence of a RT-PCR test result.

### The diagnostic challenges

The level of diagnostic certainty is significantly reduced in the absence of characteristic CT features. This is further complicated by three commonly encountered clinical scenarios – heart failure, other types of infection particularly in patients with immunosuppression, and trauma. These cases all have the potential to cause, contribute to or mask the ground glass opacification seen in COVID-19 pneumonia. The following cases illustrate the subtle features that may aid the reporting radiologist to identify the suspicious COVID-19 pneumonia from diseases that masquerade.

### COVID-19 and heart failure

**Figure 3.**
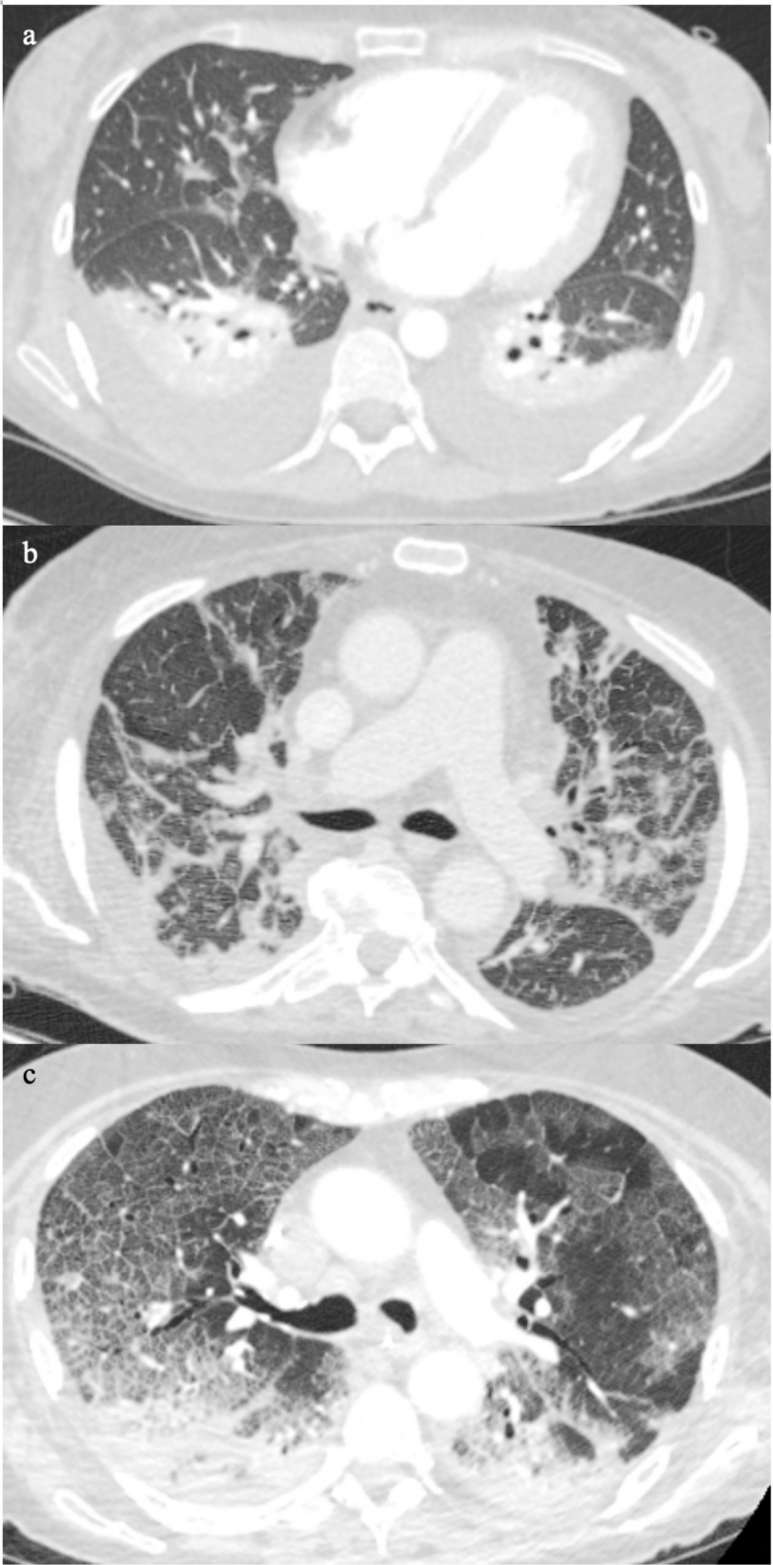
Patient A (Figure 3a) and Patient B (Figure 3b) showed different severity of interlobular septal thickening and the associated ground glass opacification is located mainly in the dependent positions. Patient C (Figure 3c) the ground glass opacification is uniformly distributed within the areas of smooth interlobular thickening resulting in the characteristic crazy-paving sign, a classic pattern on COVID-19 pneumonia.

Existing heart disease is found known to be one of the main risk factors for developing COVID-19 pneumonia.^5^ Heart failure with fluid overloading may produce symptoms and CT features that are difficult to distinguish from COVID-19 pneumonia. In this clinical scenario, three elderly patients with known heart failure presented with a history of a worsening of cough and shortness of breath without convincing COVID-19 contact. All three patients had CT pulmonary angiograms (CTPA) to exclude pulmonary embolism. The CT scans of the patients demonstrated bilateral pleural effusions with smooth interlobular septal thickening and ground glass opacification. Patient A (Figure 3a) and Patient B (Figure 3b) demonstrated different degree of interlobular septal thickening and the associated ground glass opacification is located mainly in the dependent positions. In comparison, in Patient C (Figure 3c) the ground glass opacification is more uniformly distributed within the areas of smooth interlobular thickening resulting in the crazy-paving sign, which in the right clinical context it is a supportive feature of COVID-19 pneumonia,^3,6,7^ and further supported by a positive RT-PCR test in Patient C only.

**Figure 4.**
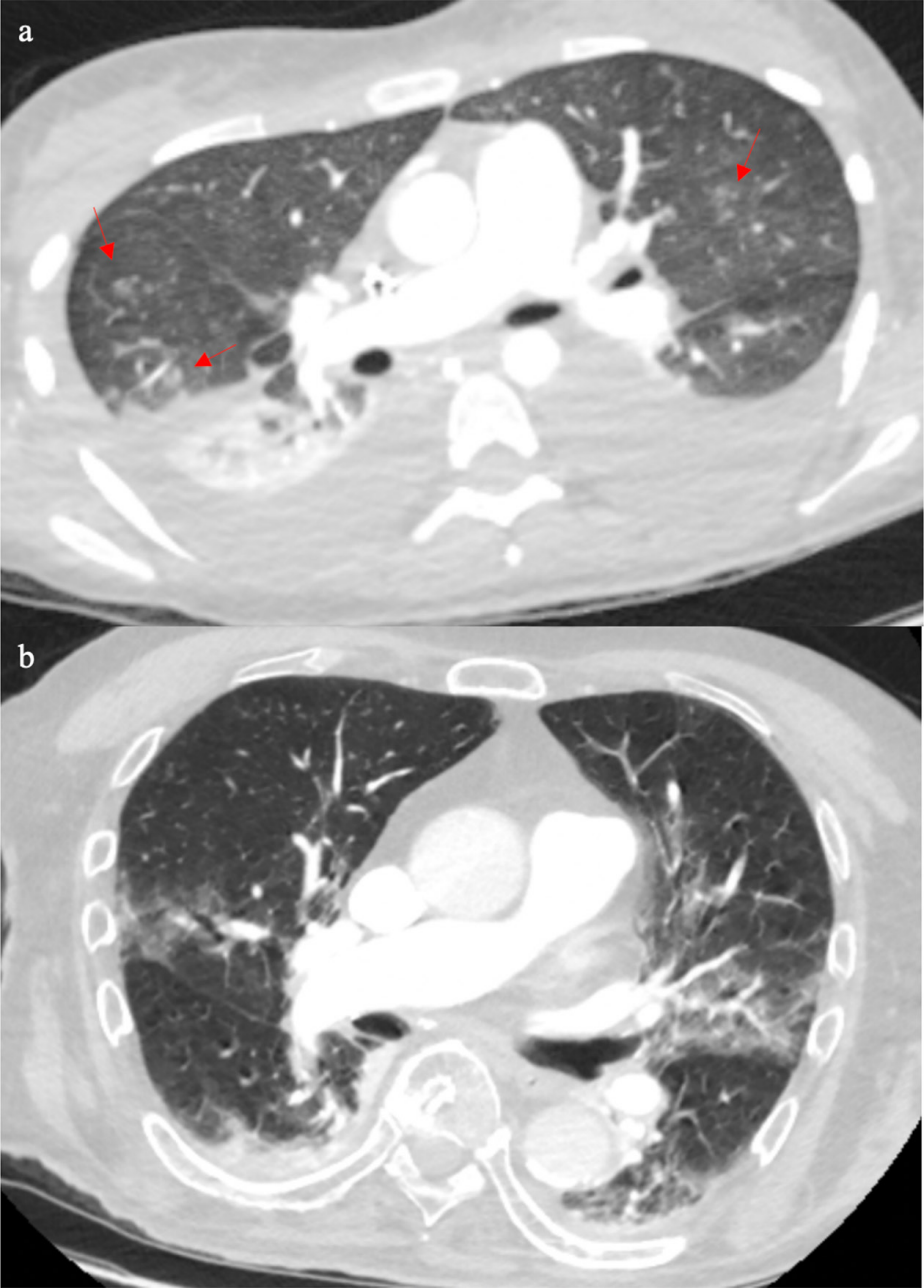
Patient D’s CT (4a) showed bilateral pleural effusions with patchy ground glass and ground glass nodularity (arrows), which is more common in bacterial infections, or if someone is immunocompromised, fungal infection is also a possibility. Patient E’s CT (4b) showed bilateral small pleural effusions with bronchocentric ground glass opacification, which in the right clinical context it could be supportive of COVID-19 pneumonia.

The defining clinical and CT features between COVID-19 pneumonia and heart failure become further complicated when there is possible superadded infection from other types of organisms. The next two patients both presented with signs and symptoms of shortness of breath and fever. Patient D’s CT demonstrated bilateral pleural effusions with patchy ground glass and ground glass nodularity (Figure 4a), which is more common in bacterial infections, or fungal infection if they are immunocompromised. In contrast, Patient E’s CT (Figure 4b) showed bilateral small pleural effusions with bronchocentric ground glass opacification. Based on recent reports, in the right clinical context bronchocentric ground glass opacification could be one of the supportive CT features of COVID-19 pneumonia.^3,6,7^ This patient was also had a positive RT-PCR test.

**Figure 5.**
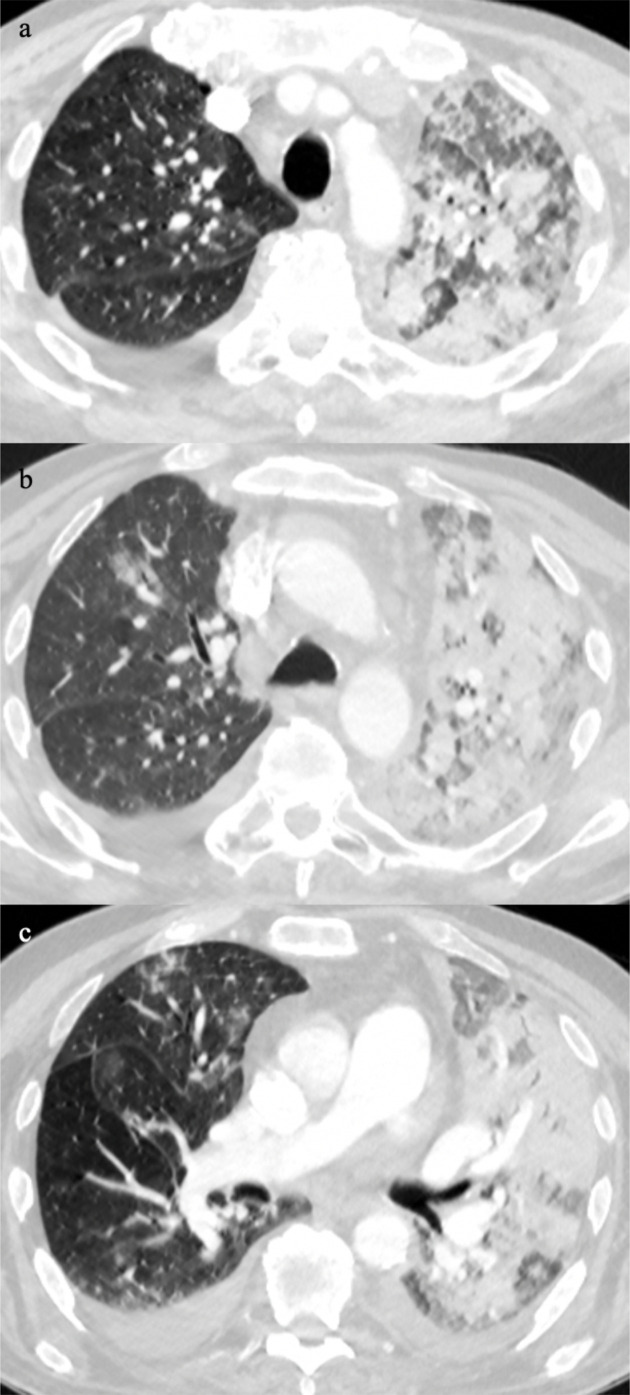
Chest CT showed predominately bronchocentric ground glass opacification in the right upper lobe and middle lobe with bilateral pleural effusions and dense consolidation on the left (5a-c).

Patient F is known to have heart failure and already has a diagnosis of COVID-19 infection from a recent RT-PCR test. He has a persistent fever and a sudden increase in his oxygen requirement. A CTPA was performed to exclude pulmonary emboli as the cause of his hypoxia, because COVID-19 infection increases the risk of thromboembolism.^8,9^ The CTPA showed predominately bronchocentric ground glass opacification in the right upper lobe and middle lobe, bilateral pleural effusions and dense consolidation on the left (Figure 5a–c). The upper zone predominance of the bronchocentric ground glass alone is indeterminant for the disease, which again emphasizes the importance of any relevant clinical information. The dense consolidation is central and unilateral affecting the left lung only, which makes it more likely to be bacterial infection than COVID-19 infection. No pulmonary embolus was present. This patient had COVID-19 and bacterial infections with fluid overload.

These cases demonstrate the complexity of separating features of concurrent COVID-19 pneumonia with heart failure and the possibility of additional infection. It is important for the radiologist to identify from the multitude of abnormalities, the individual features of each co-existing condition to help determine the diagnoses and to guide their management.

### COVID-19 and other infections

**Figure 6.**
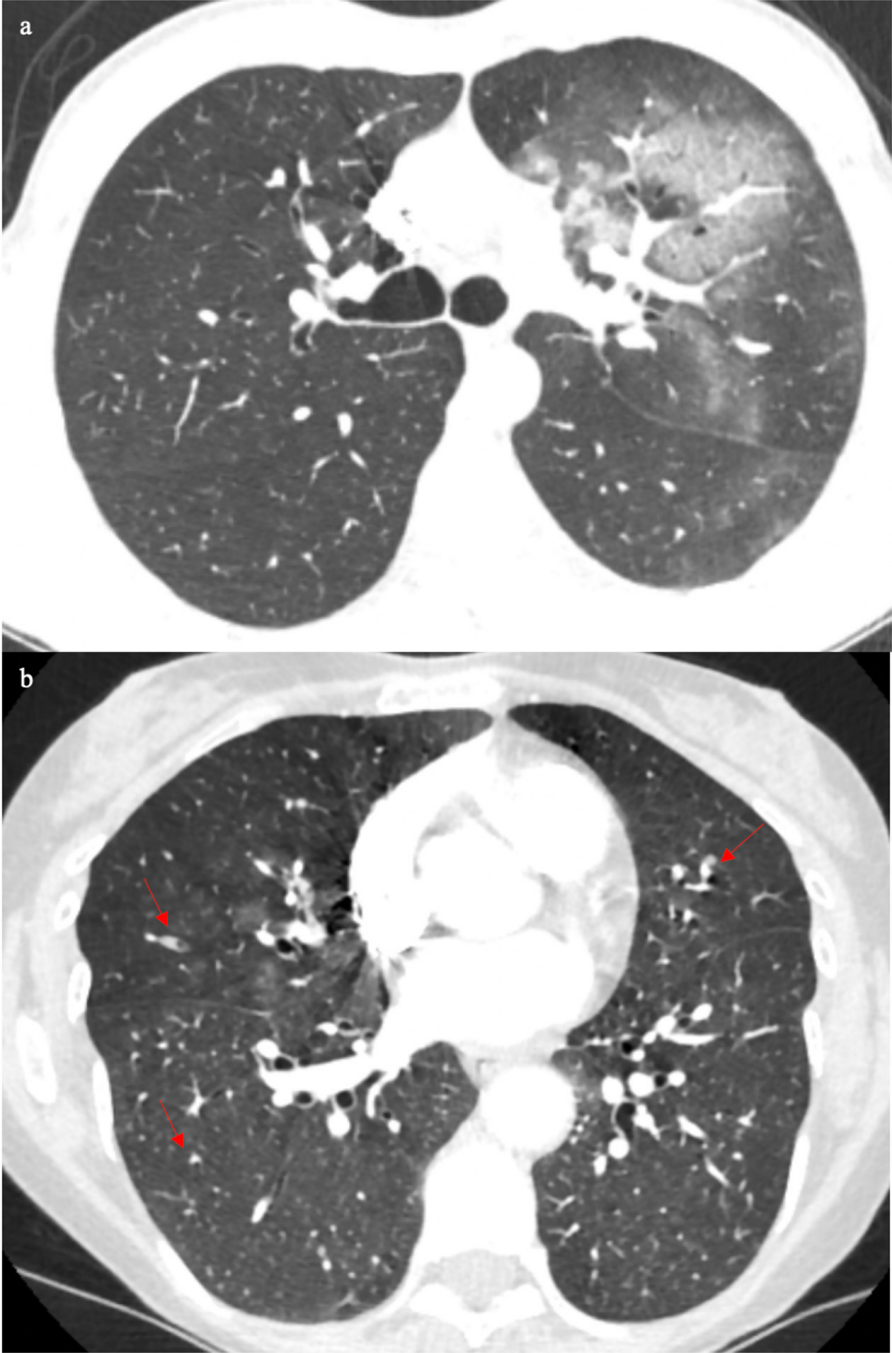
The CT showed predominantly left upper lobe central ground glass opacification (6a), which would favour lobar pneumonia, but COVID-19 infection is not completely excluded since it can also produce upper zone central ground glass opacification. Detailed inspection of the rest of the CT showed mucus plugging in the area of ground glass (Fig b, arrow), which would be consistent with bacterial infection.

During the pandemic, almost every request for chest imaging includes the possibility of COVID-19 infection. However, radiologists still need to remain vigilant in identifying other types of infection that could be misinterpreted as COVID-19. Patient G presented with cough and fever. The CT scan showed predominantly left upper lobe central ground glass opacification (Figure 6a). This feature would favour lobar pneumonia, but COVID-19 infection is not completely excluded since it can also produce upper zone central ground glass opacification. This requires detailed inspection of the rest of the CT for features of lymphadenopathy, bronchiectasis, bronchial wall thickening, and mucus plugging in the area of ground glass (Figure 6b), which increases the diagnostic confidence of bacterial infection.

**Figure 7.**
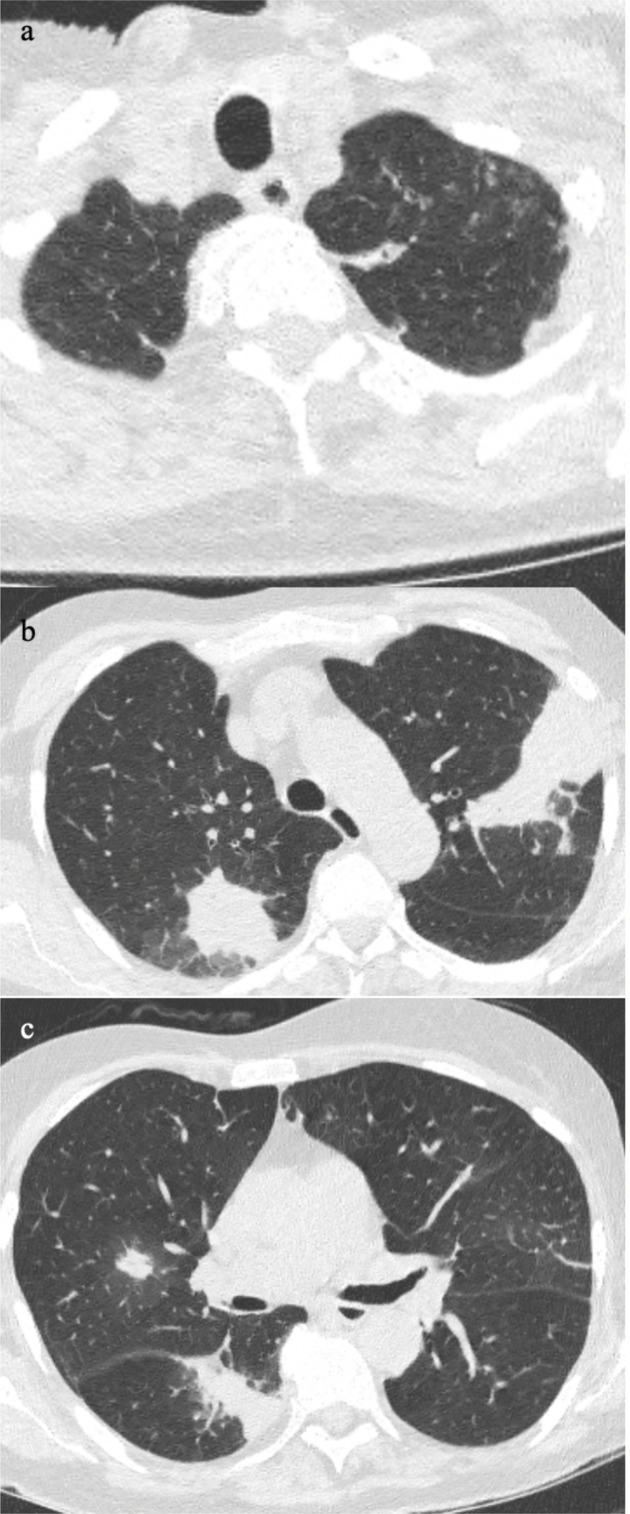
Patient has acute myeloid leukaemia and presented with neutropenic sepsis and cough. The chest CT showed mucus plugging and nodular ground glass in the left lung apex (7a), which is most likely due to bacterial infection. The mass-like consolidation in the rest of the lungs (7b-c) and a solid nodule with surrounding ground glass in the right upper lobe (7c) is more favourable of fungal infection.

**Figure 8.**
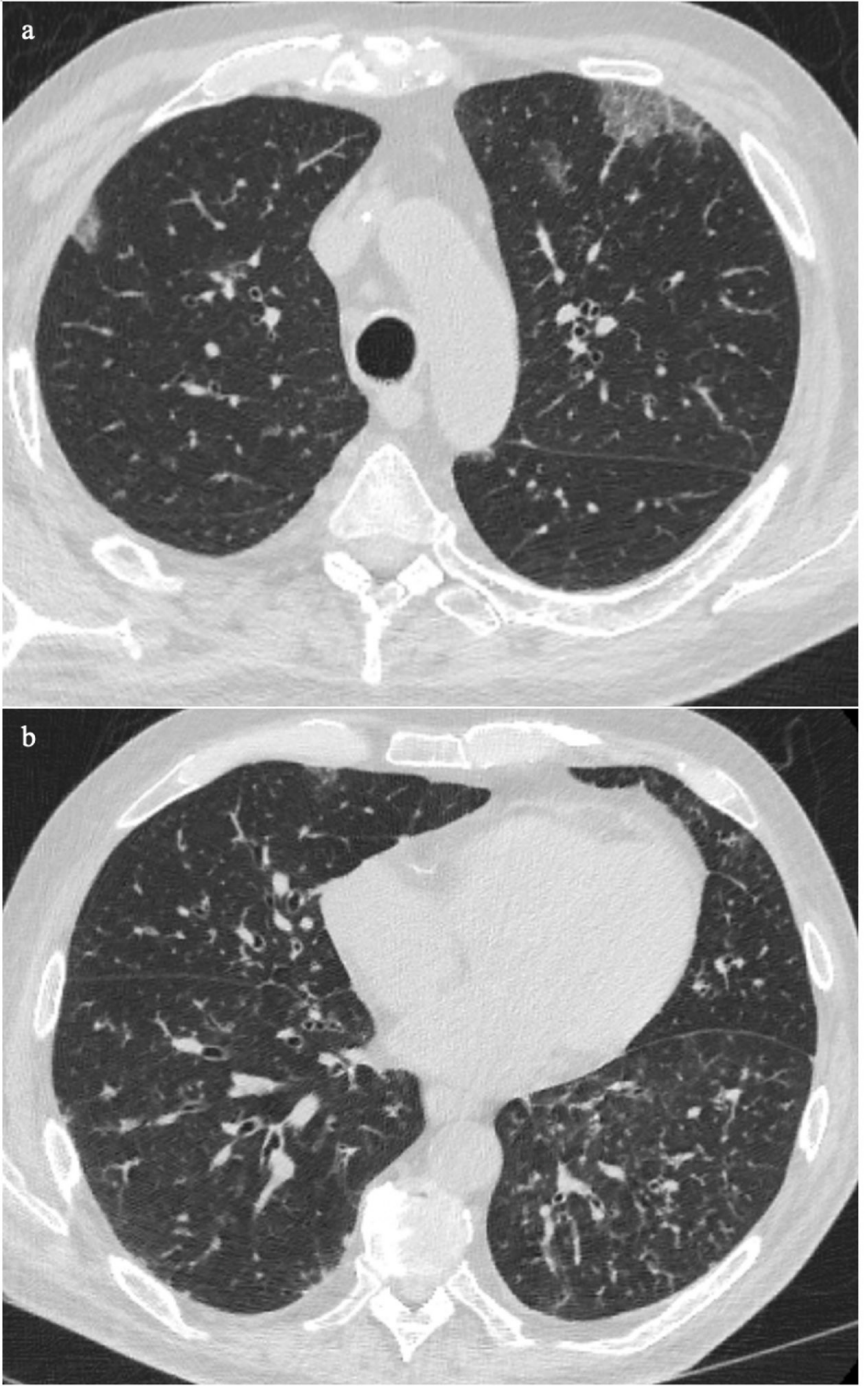
This patient is immunosuppressed with cough and fever. The CT showed peripheral crazy paving in the upper lobes, which is supportive of COVID-19 infection (8a). The bronchiectasis and mucus plugging in the lower lobes are typical of bacterial infection (8b)

The possibility of other infections occurring simultaneously or independently of COVID-19 infection is further increased in immunocompromised or immunosuppressed patients. Depending on the organism, the features may be completely different to COVID-19 infection. For example, Patient H suffers from acute myeloid leukaemia and presented with neutropaenic sepsis and cough. His CT showed mucus plugging and nodular ground glass in the left lung apex (Figure 7a), which is most likely due to bacterial infection. Furthermore, there were mass-like consolidation in the rest of the lungs (Figure 7b–c) and a solid nodule with surrounding ground glass in the right upper lobe (Figure 7c). These features are favourable for fungal infection. The next patient is immunosuppressed from treatment for post-transplant lymphoproliferative disorder (Figure 8). He presented with fever and cough. The CT showed peripheral crazy paving in the upper lobes, which is supportive of COVID-19 pneumonia Figure 8a. Whereas the bronchiectasis and mucus plugging in the lower lobes are typical of bacterial infection Figure 8b.

**Figure 9.**
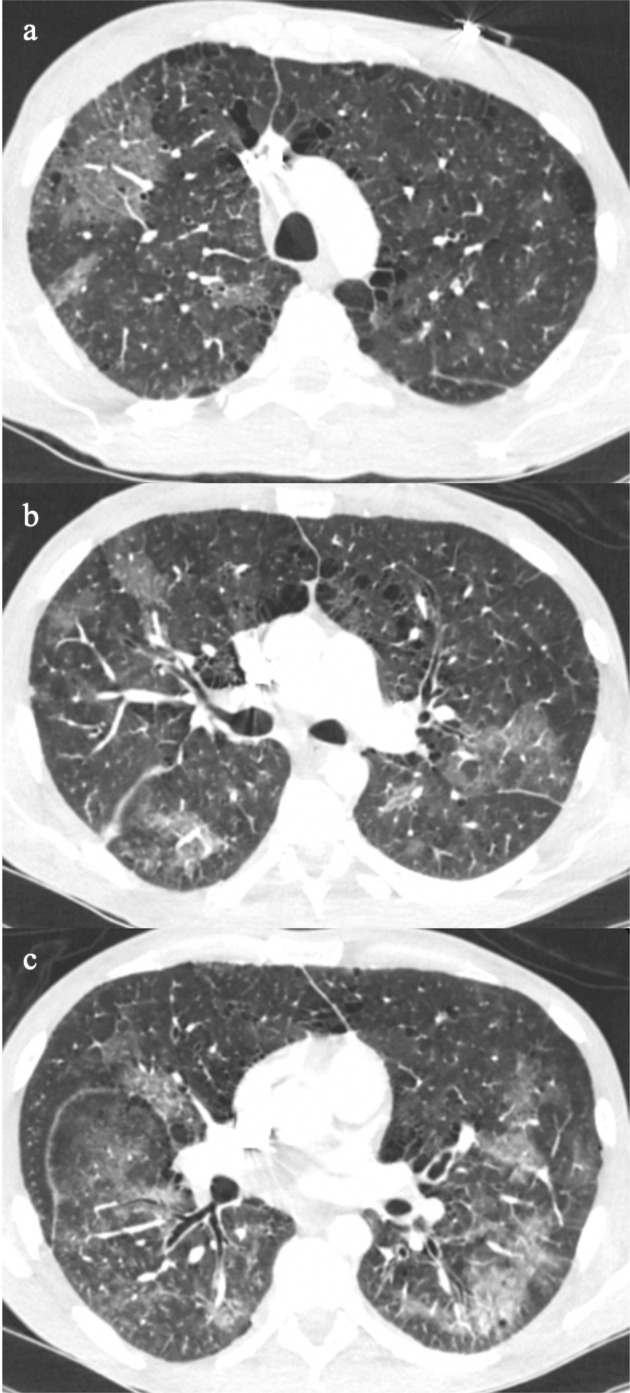
Patient was newly diagnosed with HIV and presented with fever and cough. The chest CT showed bilateral patchy ground glass opacification and slightly worse in the lower lobes (9a–c). The pattern is non-specific and could be due to various opportunistic organisms. Later, he was confirmed to have Epstein-Barr viral infection, which induced HLH. HLH, haemophagocytic lymphohistiocytosis.

There are also situations in which it is not possible to distinguish COVID-19 infection from other infections. Patient J was newly diagnosed with HIV and presented with fever and cough. The chest CT showed bilateral apical to basal patchy ground glass opacification, slightly worse in the lower lobes (Figure 9a–c). The pattern is non-specific and may be due to various opportunistic organisms especially other viruses. His blood results revealed a low haemoglobin (73 g l^−1^), low platelets (5 × 10^9^/L), and neutropaenia (non-detectable level). The cytopaenic pattern was suggestive of haemophagocytic lymphohistiocytosis (HLH). Later, he was confirmed to have Epstein-Barr viral infection, which is one of the secondary causes of HLH.^10^ He had multiple negative RT-PCR test results. This case illustrates the importance of radiologists keeping an open mind for other infections during the COVID-19 pandemic.

### COVID-19 and trauma

**Figure 10.**
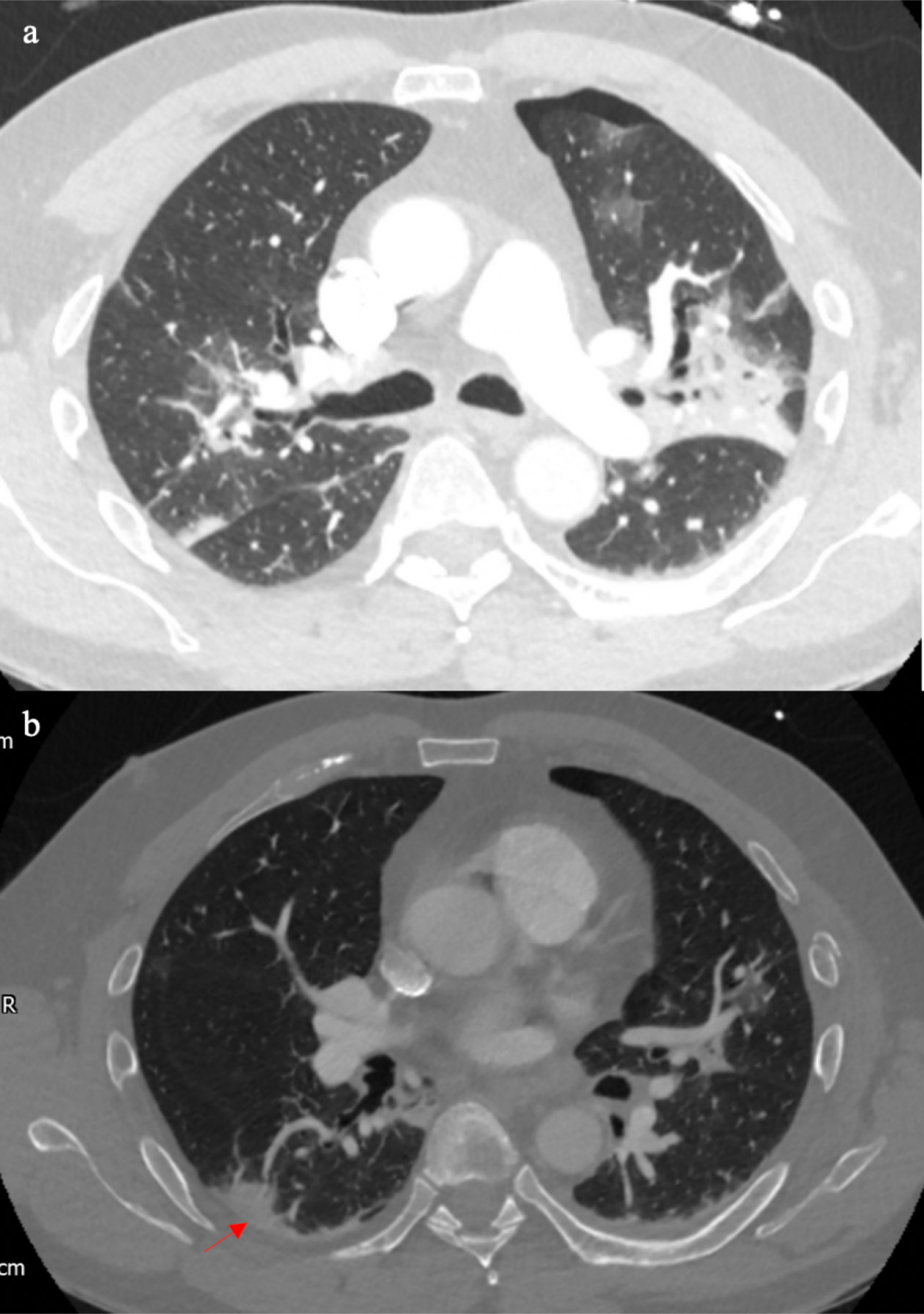
Patient with fall onto left side and trauma CT showed left-sided pneumothorax with associated left-sided rib fractures (10a–b). The centrally affected areas particularly those in the right lung were most likely from COVID-19 infection.The right posterior peripheral consolidation (arrow) could be from contre-coup injury, but infective consolidation was also possible given the absence of subpleural sparing for acute contusion.

Acute thoracic injury, especially direct impact can produce a combination of pulmonary consolidation and ground glass opacification. As a result, traumatic injuries can make the diagnosis of COVID-19 infection challenging. Patient K presented after a fall at a standing height with left-sided chest pain and shortness of breath. His trauma CT showed a left-sided pneumothorax with associated left-sided rib fractures (Figure 10a–b). There were also bilateral small areas of ground glass opacification and consolidation in the apicoposterior segment of the left upper lobe. It was possible that the peripherally affected areas were related to the trauma, but the centrally affected areas particularly those in the right lung were incompatible with the acute injuries. There was the exception of the right posterior peripheral consolidation (Figure 10b, arrow). In the context of the traumatic history, contre-coup thoracic injury was the most likely explanation but the absence of subpleural sparing was atypical for acute contusion. COVID pneumonia or bacterial infection were possibilities. Further questioning revealed that the patient had a fever for the last 14 days, and he later tested positive for COVID-19 infection only. No other organism was isolated.

A summary of the findings from Patient A to K have been presented in Table 1.

**Table 1.** Summary of findings of Patients A–K

Patient	CT findings	RT-PCR result	Diagnosis	Differential diagnosis
A&B	Interlobular septal thickening + dependent ground glass	Negative	Heart failure	
C	Interlobular septal thickening +uniform ground glass (crazy-paving sign)	Positive	COVID pneumonia	
D	Patchy ground glass & ground glass nodularity	Negative	Bacterial infection	Fungal infection if immunocompromised
E	Bronchocentric ground glass	Positive	COVID pneumonia	Vasculitis
F	Upper zone predominant ground glass & unilateral dense consolidation	Negative	Bacterial infection	
G	Upper zone central ground glass	Negative	Bacterial infection	COVID pneumonia
H	Mucus plugging, nodular ground glass Mass-like consolidation Solid nodule with surrounding ground glass	Negative	Bacterial infection Fungal infection	
I	Peripheral crazy paving	Positive	COVID pneumonia	
J	Patchy ground glass	Negative	Non-specific; Epstein-Barr viral infection (Require clinical information)	
K	Peripheral ground glass and/or consolidation with traumatic injuries Central ground glass & peripheral consolidation without subpleural sparing.	Positive	Traumatic	COVID pneumonia Bacterial infection

RT-PCR, reverse transcription polymerase chain reaction.

## Conclusion

The classic CT patterns of COVID-19 pneumonia have been well-reported and are now more readily recognised by radiologists. However, a confident diagnosis of COVID-19 pneumonia can become difficult when there are concurrent conditions which may result in similar patterns of disease. In these cases, the imaging must be scrutinised carefully. During the peak and recovery phases of the pandemic, radiologists must remain vigilant to enable the correct diagnosis of COVID-19 pneumonia to be made and distinguished from its mimics.

## Learning points

1. All radiologists should be aware of the typical and atypical CT patterns of COVID-19 pneumonia.
2. Pre-existing conditions and traumatic thoracic injuries can make the diagnosis challenging.
3. Other types of infection particularly viral can be difficult to distinguish from COVID-19 pneumonia.
4. Subtle imaging clues should be scrutinised carefully to identify the non-COVID-19 conditions that may mask or contribute to similar disease pattern.
